# Proton Pump Inhibitors Prescribing Behaviors and Rationalization Strategies Among Healthcare Providers in Southeast Asia

**DOI:** 10.1002/prp2.70274

**Published:** 2026-06-02

**Authors:** Duc Trong Quach, Tien Manh Huynh, Augusto Jose G. Galang, Hang Viet Dao, Sakkarin Chirapongsathorn, Phuripong Kijdamrongthum, Rahela Ambaras Khan, Andrew Ming‐Liang Ong, Wee Kooi Cheah, Kalwinder Singh Khaira, So Fie Tan, Jose Sollano, Ari Fahrial Syam, Tju Siang Chua, Somchai Leelakusolvong, Yeong Yeh Lee

**Affiliations:** ^1^ Department of Internal Medicine and Gastro‐Hepato Integrated Research Team (GHIRT‐002.TCM2025) University of Medicine and Pharmacy at Ho Chi Minh City Ho Chi Minh City Vietnam; ^2^ Department of Gastroenterology Nhan Dan Gia Dinh Hospital Ho Chi Minh City Vietnam; ^3^ Section of Gastroenterology, Department of Medicine Angeles University Foundation Medical Center Angeles City Philippines; ^4^ Internal Medicine Faculty, Endoscopy Centre Hanoi Medical University, Hanoi Medical University Hospital Hanoi Vietnam; ^5^ Division of Gastroenterology and Hepatology, Department of Medicine Phramongkutklao Hospital and College of Medicine Bangkok Thailand; ^6^ Division of Gastroenterology, Department of Internal Medicine Chiang Mai University Chiang Mai Thailand; ^7^ Department of Pharmacy Hospital Kuala Lumpur, Ministry of Health Kuala Lumpur Malaysia; ^8^ Department of Gastroenterology and Hepatology Singapore General Hospital Singapore Singapore; ^9^ Department of Medicine and Clinical Research Center Hospital Taiping Taiping Perak Malaysia; ^10^ Department of Medicine Sarawak General Hospital Kuching Sarawak Malaysia; ^11^ Regional Medical Science Reckitt Benckiser (M) Sdn. Bhd Kuala Lumpur Malaysia; ^12^ Faculty of Medicine and Surgery University of Santo Tomas Manila Philippines; ^13^ Division of Gastroenterology, Department of Internal Medicine Faculty of Medicine Universitas Indonesia—Cipto Mangunkusumo General Hospital Jakarta Indonesia; ^14^ AliveoMedical Singapore Singapore; ^15^ Division of Gastroenterology, Department of Medicine Faculty of Medicine Siriraj Hospital, Mahidol University Bangkok Thailand; ^16^ Siriraj GI Endoscopy Center, Siriraj Hospital Bangkok Thailand; ^17^ School of Medical Sciences Universiti Sains Malaysia Kota Bharu Malaysia; ^18^ Department of Internal Medicine, Faculty of Medicine, Dr. Soetomo Teaching Hospital Universitas Airlangga Surabaya Indonesia

**Keywords:** deprescription, drug prescriptions, health personnel, proton pump inhibitors, Southeastern Asia

## Abstract

Despite widespread use, real‐world evidence regarding proton pump inhibitor (PPI) prescribing and rationalization practices in Southeast Asia (SEA) remains limited; therefore, we aimed to characterize current prescribing behaviors and rationalization strategies among healthcare professionals in the region. The SEA PPI Rationalisation Working Group developed and disseminated an online questionnaire via professional networks across SEA to collect data on respondent demographics, PPI prescribing practices, and rationalization approaches; instrument reliability testing produced a Cronbach's *α* of 0.799 and content validity was confirmed by a panel of 10 gastroenterology experts with complete agreement on relevance and clarity (I‐CVI, kappa, SCVI/Ave = 1.0). K‐mode clustering was applied to delineate prescribing patterns. Of 869 responses received, 763 valid entries were analyzed (response rate 87.8%), 49% of whom were gastroenterologists. PPIs were most frequently prescribed for gastroesophageal reflux disease (67.8%) and peptic ulcer disease (43.8%); 48.9% of respondents reported long‐term PPI use, while 59.2% reported reassessment of indications at every follow‐up visit. Common rationalization strategies comprised dose tapering (46.8%), on‐demand administration (45.9%), and step‐down to antacids or alginates (44.8%). Cluster analysis identified two distinct prescriber profiles: proactive prescribers (*n* = 326, 42.7%), who adhered to clinical guidelines, implemented individualized dosing, and routinely reassessed therapy; and conservative prescribers (*n* = 437, 57.3%), who employed PPIs more restrictively and engaged less frequently in rationalization. The considerable variability in PPI prescribing and rationalization practices across SEA underscores the need for a regional consensus to promote evidence‐based practice.

## Graphical abstract

Long‐term proton pump inhibitor (PPI) therapy raises significant concerns regarding cost‐effectiveness and adverse effects, necessitating effective deprescribing strategies. An evaluation of 763 Southeast Asian healthcare providers revealed two distinct prescriber phenotypes, where a proactive cluster utilized diverse, guideline‐based rationalization strategies and frequent reassessment, while a conservative majority favored fixed standard dosing, suggesting that targeted stewardship efforts focusing on non‐gastroenterologists and non‐academic settings are warranted to optimize regional PPI prescribing.

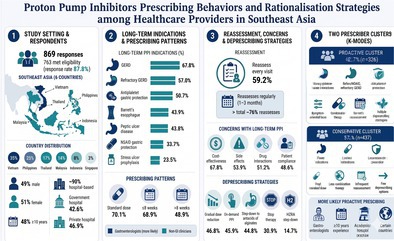

## 1 Introduction

Proton pump inhibitors (PPIs) are the mainstay in the treatment of acid‐related gastrointestinal disorders. The clinical efficacy of these drugs has led to widespread global prescription. Nevertheless, the inappropriate and prolonged use of PPIs has become a growing concern. Several studies have reported significantly inappropriate prescribing rates in primary care and hospital inpatients [1, 2]. In Southeast Asia (SEA), this issue is particularly pronounced, with several studies in the region reporting inappropriate PPI prescribing rates ranging from 40% to 60% [3, 4, 5]. Long‐term PPI use has raised concerns about its potential association with a spectrum of adverse clinical outcomes, including *Clostridioides difficile* infection, small intestinal bacterial overgrowth, chronic kidney disease, bone fractures, and micronutrient deficiencies, although the current level of evidence remains low [6, 7]. However, acid‐related gastrointestinal disorders in Asian populations are somewhat different from those in their Western counterparts. Notably, although the prevalence of gastroesophageal reflux disease (GERD) is increasing in the region, cases of erosive reflux disease are generally mild, and endoscopic complications remain uncommon [8]. These scenarios typically do not necessitate long‐term PPI maintenance therapy. These findings suggest that PPI prescribing patterns in clinical practice across SEA may differ substantially from those observed in Western healthcare settings. However, despite these differences, regional data remain sparse and fragmented, and there is still no unified consensus on deprescribing protocols tailored to the region [9]. From a clinical pharmacology perspective, PPIs exemplify a class of drugs with high global exposure, frequent overprescribing, and growing concern about potential long‐term harms and costs, prompting calls for better prescribing quality and deprescribing strategies [10]. Yet how prescribers in SEA actually use and rationalize PPIs in routine practice remains poorly defined. In this context, the present study aimed to evaluate current PPI prescribing behaviors and rationalization strategies among healthcare providers across SEA, with the ultimate goal of informing evidence‐based, regionally tailored recommendations to optimize the appropriate use of PPIs within the region's varied and dynamically changing healthcare environment.

## 2 Methods

### 2.1 Survey Development and Validation

The questionnaire was developed by the SEA PPI Rationalisation Working Group. The content development was based on three core expert insights from the group and review of existing literature using predefined search criteria. Initial iterations of the newly developed questionnaire were then distributed to three other experts for comments and revised accordingly. The final English language‐based questionnaire comprises 13 structured items across three sections: (1) demographic and professional background; (2) PPI prescribing habits and perceptions; and (3) PPI rationalization strategies (Data S1). Content validity was evaluated by a panel of 10 regional experts, with each item assessed via the content validity index (CVI) [11].

A pilot test involving 30 healthcare professionals was conducted to evaluate clarity, item sequence, and overall comprehensibility. The participants were recruited through professional networks of the SEA PPI Rationalisation Working Group using a convenience sampling approach. They included healthcare professionals involved in the management of acid‐related gastrointestinal disorders from participating SEA countries. The purpose of the pilot test was to assess the clarity and usability of the questionnaire rather than to achieve statistical representativeness.

### 2.2 Online Study Design and Participants

The survey was decided by the working group to distribute online and using cross‐sectional design. It was distributed using Survey Monkey (website: https://www.surveymonkey.com/) between July and August 2025 in six Southeast Asian countries: Indonesia, Malaysia, the Philippines, Singapore, Thailand, and Vietnam. The participants were healthcare professionals who were members of national societies in gastroenterology, internal medicine, geriatrics, or pharmacy. The eligibility criteria were as follows: (1) active membership in at least one of these professional societies and (2) provision of informed consent by agreeing to participate at the beginning of the survey. The respondents were recruited through the official communication channels of the respective societies. In addition, targeted email invitations were sent by core members of the SEA PPI Rationalisation Working Group, many of whom also served as executive members of the participating national societies. This recruitment strategy aimed to ensure diverse professional representations and enhance response validity across countries. Only fully completed questionnaires were included in the final dataset, and duplicate submissions identified through identical IP addresses were excluded. The study adheres to the STROBE guidelines for reporting observational studies [12].

### 2.3 Statistical Analysis

Descriptive statistics were used to summarize the demographic and survey response data including categorical variables (frequencies and percentages) and continuous variables (means with standard deviations or medians with interquartile ranges).

Free‐text answers provided under “Other (please specify)” were reviewed and coded by two independent investigators, with discrepancies resolved by consensus. Responses that matched an existing option were recategorized accordingly, whereas remaining answers were grouped into descriptive subcategories or, when infrequent and heterogeneous, reported only qualitatively and excluded from the main quantitative analyses.

Group comparisons were performed across two key subgroups: (i) gastroenterology (GI) versus non‐GI healthcare professionals (all other specialties) and (ii) high‐level healthcare experience (HE; defined as ≥ 10 years in clinical practice) versus low‐level experience (LE; < 10 years). For comparisons involving categorical variables, either Pearson's chi‐square test or Fisher's exact test was applied, as deemed appropriate.

K‐mode clustering, an unsupervised machine learning algorithm suitable for categorical data, was used to explore latent behavioral patterns [13]. The respondents were grouped based on similarities across multiple survey domains, including indications for long‐term PPI use, deprescribing strategies, and adjunctive therapies. In this survey, long‐term PPI use was defined as treatment beyond 8 weeks, reflecting the usual 4–8‐week course recommended for uncomplicated GERD and peptic ulcer disease, after which ongoing therapy is generally considered maintenance or long‐term use in clinical studies and expert guidance [14].

The optimal number of clusters was determined using the elbow method based on within‐cluster dissimilarity. To identify predictors of proactive prescribing behavior, univariate logistic regression analyses were conducted to estimate crude odds ratios (ORs) with corresponding 95% confidence intervals (CIs). Variables with *p* < 0.05 or those considered clinically meaningful were subsequently included in a multivariable logistic regression model to estimate adjusted ORs and 95% CIs. All analyses were performed via R version 4.4.0 (R Foundation for Statistical Computing, Vienna, Austria). Statistical significance was defined as a two‐sided *p* < 0.05.

### 2.4 Ethical Considerations

The study obtained approval from the Institutional Review Board of the University of Medicine and Pharmacy at Ho Chi Minh City, Vietnam (approval number: 2579/DHYD‐HDDD). It was conducted in accordance with the ethical principles outlined in the Declaration of Helsinki. The authors confirm that the PI for this paper is DTQ and that he had direct clinical responsibility for this survey.

## 3 Results

### 3.1 Survey Validation

The newly developed survey achieved a scale‐CVI/average of 1.0, indicating excellent content validity. From pilot testing, the final questionnaire demonstrated good internal consistency, with a Cronbach's *α* of 0.799.

### 3.2 Cross‐Sectional Online Survey

**FIGURE 1:**
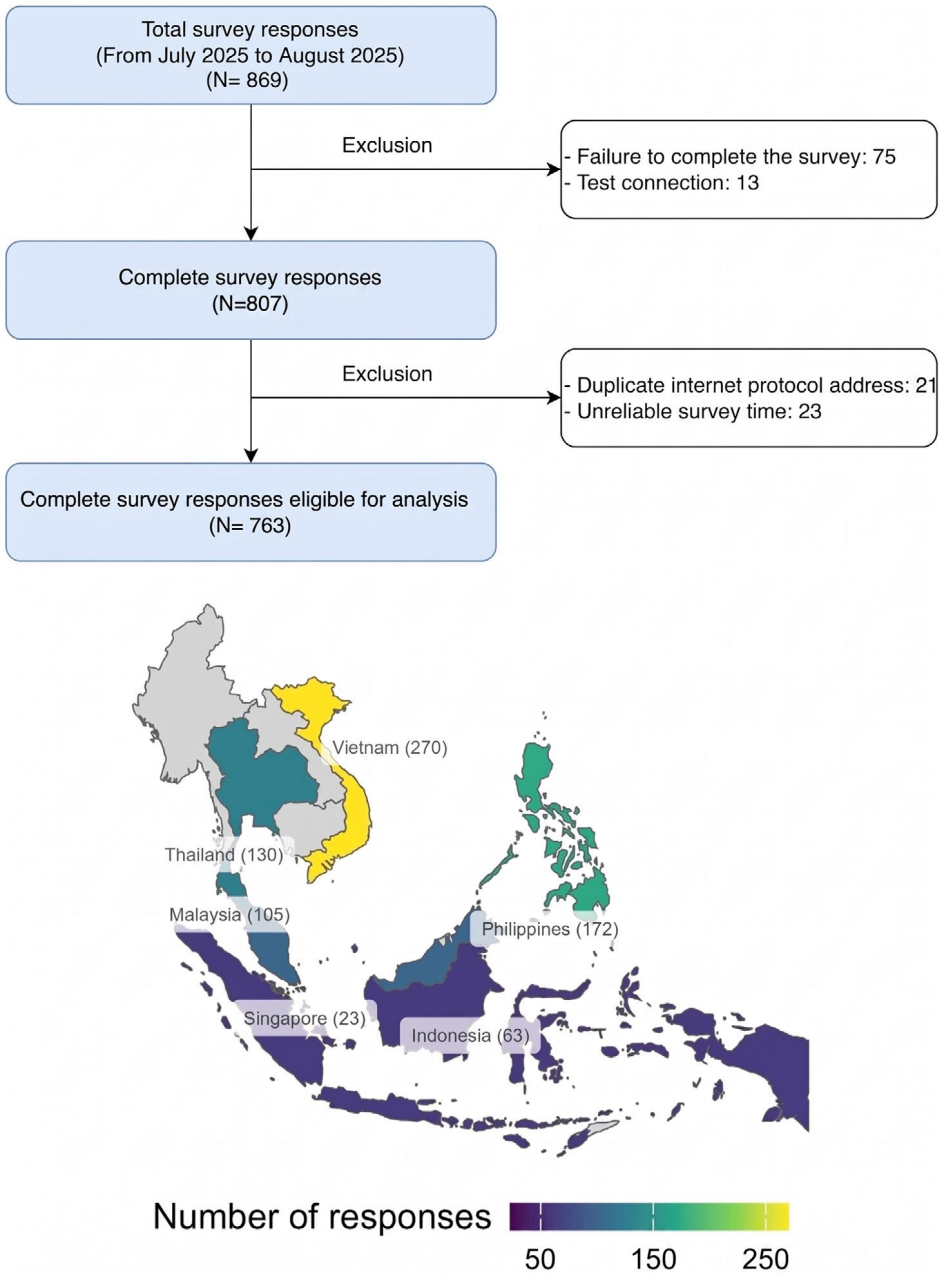
Study flow and geographic distribution of survey respondents.

A total of 869 responses were collected via the online survey platform, of which 763 met the eligibility criteria and were included in the final analysis (Figure 1). The baseline characteristics of participants are presented in Table 1. Nearly half of the participants were GIs and half were with more than 10 years of clinical experience (49.4% and 48.3%, respectively). The majority of participants were hospital‐based, with 42.6% working in government hospitals and 46.9% working in private hospitals.

**TABLE 1:** Baseline characteristics of survey respondents (*N* = 763).

Characteristic	*n* (%)
Specialty	
Gastroenterology	377 (49.4)
General practitioner/family physician	145 (19.0)
Geriatric medicine	10 (1.3)
Internal medicine	123 (16.1)
Obstetrics and gynecology	2 (0.3)
Otolaryngology	68 (8.9)
Pharmacist	28 (3.7)
Other^a^	10 (1.3)
Year in practice	
< 5 years	180 (23.6)
5–10 years	214 (28.0)
11–20 years	223 (29.3)
> 20 years	146 (19.1)
Practice setting	
Hospital‐based (government)	325 (42.6)
Hospital‐based (private)	358 (46.9)
Private practice	160 (21.0)
Academic/research	91 (11.9)
Country	
Vietnam	270 (35.4)
Philippines	172 (22.6)
Singapore	23 (2.9)
Thailand	130 (17.0)
Malaysia	105 (13.8)
Indonesia	63 (8.3)
Proportion of prescriptions containing PPIs in the past month	
≤ 10%	55 (7.2)
≥ 51%	170 (22.3)
11%–20%	102 (13.4)
21%–30%	169 (22.1)
31%–40%	143 (18.7)
41%–50%	124 (16.3)

^a^ Occupational medicine (3), dermatology (2), emergency medicine (2), ophthalmology (1), pediatrics (1), traditional medicine (1).

The most frequently reported indications for long‐term PPI use were GERD (67.8%), refractory GERD (57.0%), and antiplatelet therapy‐induced gastric protection (50.7%) (Table 2). GIs were more likely than non‐GIs to prescribe PPIs for Barrett's esophagus (58.9% vs. 29.3%, *p* < 0.001), refractory GERD (68.4% vs. 45.9%, *p* < 0.001), antiplatelet therapy‐induced gastric protection (62.3% vs. 37.7%, *p* < 0.001), and nonsteroidal anti‐inflammatory drug (NSAID)‐induced gastric protection (38.2% vs. 29.3%, *p* = 0.01). Interestingly, HEs were more likely than their LE counterparts to prescribe PPIs for chronic steroid therapy‐induced gastric protection (35.2% vs. 26.4%, *p* = 0.008).

**TABLE 2:** Common indications for long‐term proton pump inhibitor use.

Indication	All	Speciality		*p* *	Experience		*p* *
	*n* = 763 (%)	Non‐GI, *n* = 386 (%)	GI, *n* = 377 (%)		LE, *n* = 394 (%)	HE, *n* = 369 (%)	
Antiplatelet therapy‐induced gastric protection	387 (50.7)	152 (39.3)	235 (62.3)	**< 0.001**	200 (50.8)	187 (50.7)	0.982
Nonsteroidal anti‐inflammatory drug‐induced gastric protection	257 (33.7)	113 (29.3)	144 (38.2)	**0.01**	116 (29.4)	141 (38.2)	**0.01**
Chronic steroid therapy‐induced gastric protection	234 (30.7)	112 (29.0)	122 (32.4)	0.316	104 (26.4)	130 (35.2)	**0.008**
Stress ulcer prophylaxis in critically ill patients	179 (23.5)	102 (26.4)	77 (20.4)	**0.05**	88 (22.3)	91 (24.7)	0.449
Gastroesophageal reflux disease	517 (67.8)	278 (72.0)	239 (63.4)	**0.01**	254 (64.5)	263 (71.3)	**0.04**
Oesophagitis	197 (25.8)	93 (24.1)	104 (27.6)	0.172	80 (20.3)	117 (31.7)	**< 0.001**
Refractory gastroesophageal reflux disease	435 (57.0)	177 (45.9)	258 (68.4)	**< 0.001**	207 (52.5)	228 (61.8)	**0.01**
Barrett's esophagus	335 (43.9)	113 (29.3)	222 (58.9)	**< 0.001**	164 (41.6)	171 (46.3)	0.189
Extraoesophageal reflux/laryngopharyngeal reflux	253 (33.2)	160 (41.5)	93 (24.7)	**< 0.001**	115 (29.2)	138 (37.4)	**0.016**
Functional dyspepsia	184 (24.1)	89 (23.1)	95 (25.2)	0.49	95 (24.1)	89 (24.1)	0.998
Peptic ulcer disease	334 (43.8)	179 (46.4)	155 (41.1)	0.143	166 (42.1)	168 (45.5)	0.345

*Note:* Boldface values highlight statistically significant findings (*p* < 0.05).

Abbreviations: GI, gastroenterologist; HE, high experience; LE, low experience; Non‐GI, non‐gastroenterologist.

* Pearson's chi‐square test was used to compare proportions (Fisher's exact test was applied where appropriate).

Regarding patterns of PPI prescribing, the majority of respondents used standard, guideline‐recommended dosages (70.1%) and usually for short‐term use (68.9%) (Table 3). GIs were more likely than non‐GIs to prescribe combination therapy (70.8% vs. 49.0%, *p* < 0.001), to titrate dosages based on symptoms (56.5% vs. 44.0%, *p* < 0.001), and to use PPIs long‐term (54.9% vs. 43.0%, *p* < 0.001). Similarly, compared to LE providers, HE providers used combination therapy (65.9% vs. 54.1%, *p* < 0.001), symptom‐based titration (55.3% vs. 45.4%, *p* = 0.008), and long‐term treatment (53.4% vs. 44.7%, *p* = 0.016) more frequently.

**TABLE 3:** Patterns of PPI prescription or recommendation.

Pattern	All	Speciality		*p* *	Experience		*p* *
	*N* = 763 (%)	Non‐GI, *n* = 386 (%)	GI, *n* = 377 (%)		LE, *n* = 394 (%)	HE, *n* = 369 (%)	
Standard, guideline‐recommended dosage for the specific PPI	535 (70.1)	261 (67.6)	274 (72.7)	0.13	271 (68.8)	264 (71.5)	0.405
Short‐term use (< 8 weeks) for a defined duration based on indication	526 (68.9)	251 (65.0)	275 (72.9)	**0.02**	260 (66.0)	266 (72.1)	0.069
Combination therapy with other medications (e.g., *Helicobacter pylori* eradication)	456 (59.8)	189 (49.0)	267 (70.8)	**< 0.001**	213 (54.1)	243 (65.9)	**< 0.001**
Adjustable dosage titrated based on symptom severity or response	383 (50.2)	170 (44.0)	213 (56.5)	**< 0.001**	179 (45.4)	204 (55.3)	**0.008**
Long‐term use (beyond 8 weeks) for chronic conditions or maintenance therapy	373 (48.9)	166 (43.0)	207 (54.9)	**< 0.001**	176 (44.7)	197 (53.4)	**0.016**
As‐needed basis when symptoms arise	296 (38.8)	126 (32.6)	170 (45.1)	**< 0.001**	145 (36.8)	151 (40.9)	0.243

*Note:* Boldface values highlight statistically significant findings (*p* < 0.05).

Abbreviations: GI, gastroenterologist; HE, high experience; LE, low experience; Non‐GI, non‐gastroenterologist.

* Pearson's chi‐square test was used to compare proportions (Fisher's exact test was applied where appropriate).

The most common concerns for long‐term PPI use were cost‐effectiveness (67.8%), side effects (53.9%), and potential drug interactions (51.2%). GIs expressed significantly greater concern on side effects than the non‐GIs (60.7% vs. 47.2%, *p* < 0.001). HE providers reported more concerns about side effects (60.7% vs. 47.5%, *p* < 0.001) and patient compliance (55.0% vs. 42.6%, *p* < 0.001) than their LE counterparts (Table 4). More than half of participants (59.2%) reported reassessing the indication for PPI use at every follow‐up visit. The patterns of reassessment did not differ significantly either between GIs and non‐GIs or between HE and LE healthcare providers (Table S1).

**TABLE 4:** Concerns of participants when prescribing or recommending long‐term PPI therapy.

Concern	All	Speciality		*p* ^a^	Experience		*p* ^a^
	*N* = 763 (%)	Non‐GI, *n* = 386 (%)	GI, *n* = 377 (%)		LE, *n* = 394 (%)	HE, *n* = 369 (%)	
Cost‐effectiveness	517 (67.8)	267 (69.2)	250 (66.3)	0.398	256 (65.0)	261 (70.7)	0.089
Side effects	411 (53.9)	182 (47.2)	229 (60.7)	**< 0.001**	187 (47.5)	224 (60.7)	**< 0.001**
Drug interactions	391 (51.2)	204 (52.8)	187 (49.6)	0.370	192 (48.7)	199 (53.9)	0.151
Efficacy	377 (49.4)	201 (52.1)	176 (46.7)	0.137	202 (51.3)	175 (47.4)	0.289
Patients' compliance	371 (48.6)	199 (51.6)	172 (45.6)	0.171	168 (42.6)	203 (55.0)	**< 0.001**
Over‐the‐counter indications	101 (13.2)	51 (13.2)	50 (13.3)	0.984	52 (13.2)	49 (13.3)	0.974
Other^b^	5 (0.7)	1 (0.3)	4 (1.1)	0.170	1 (0.3)	4 (1.1)	0.156

*Note:* Boldface values highlight statistically significant findings (*p* < 0.05).

Abbreviations: GI, gastroenterologist; HE, high experience; LE, low experience; Non‐GI, non‐gastroenterologist.

^a^ Pearson's chi‐square test was used to compare proportions (Fisher's exact test was applied where appropriate).

^b^ No concern (2), pill burden (1), and not available (2).

The most common strategies for deprescribing PPIs included gradual dose reduction (46.8%), switching to on‐demand use (45.9%), and stepping down to antacids/alginates (44.8%) (Table 5). Compared with non‐GIs, GIs were more likely to use gradual tapering (52.5% vs. 41.2%, *p* = 0.002) and on‐demand regimens (55.7% vs. 36.3%, *p* < 0.001). HE providers were more likely to step down to H2‐receptor antagonists than their LE counterparts (17.3% vs. 12.2%, *p* = 0.044).

**TABLE 5:** Strategies for deprescribing PPIs.

Strategy	All, *N* = 763 (%)	Speciality			Experience		
		Non‐GI, *n* = 386 (%)	GI, *n* = 377 (%)	*p* *	LE, *n* = 394 (%)	HE, *n* = 369 (%)	*p* *
Gradual dose reduction over time	357 (46.8)	159 (41.2)	198 (52.5)	**0.002**	180 (45.7)	177 (48.0)	0.528
Switch to on‐demand PPI use	350 (45.9)	144 (36.3)	210 (55.7)	**< 0.001**	167 (42.4)	183 (49.6)	**0.046**
Step down to antacids or alginates	342 (44.8)	187 (48.4)	155 (41.1)	**0.042**	178 (45.2)	164 (44.4)	0.839
Immediate discontinuation of PPI	236 (30.9)	121 (31.3)	115 (30.5)	0.801	115 (29.2)	121 (32.8)	0.282
Step down to H2 receptor antagonists (H2RA)	112 (14.7)	56 (14.5)	56 (14.9)	0.892	48 (12.2)	64 (17.3)	**0.044**
Other	3 (0.4)	0 (0)	3 (0.8)	0.079	1 (0.3)	2 (0.5)	0.525

*Note:* Other—depending on initial indication (2) and no need (1). Boldface values highlight statistically significant findings (*p* < 0.05).

* Pearson's chi‐square test was used to compare proportions (Fisher's exact test was applied where appropriate).

### 3.3 Cluster Analysis of PPI Prescribing Patterns

**FIGURE 2:**
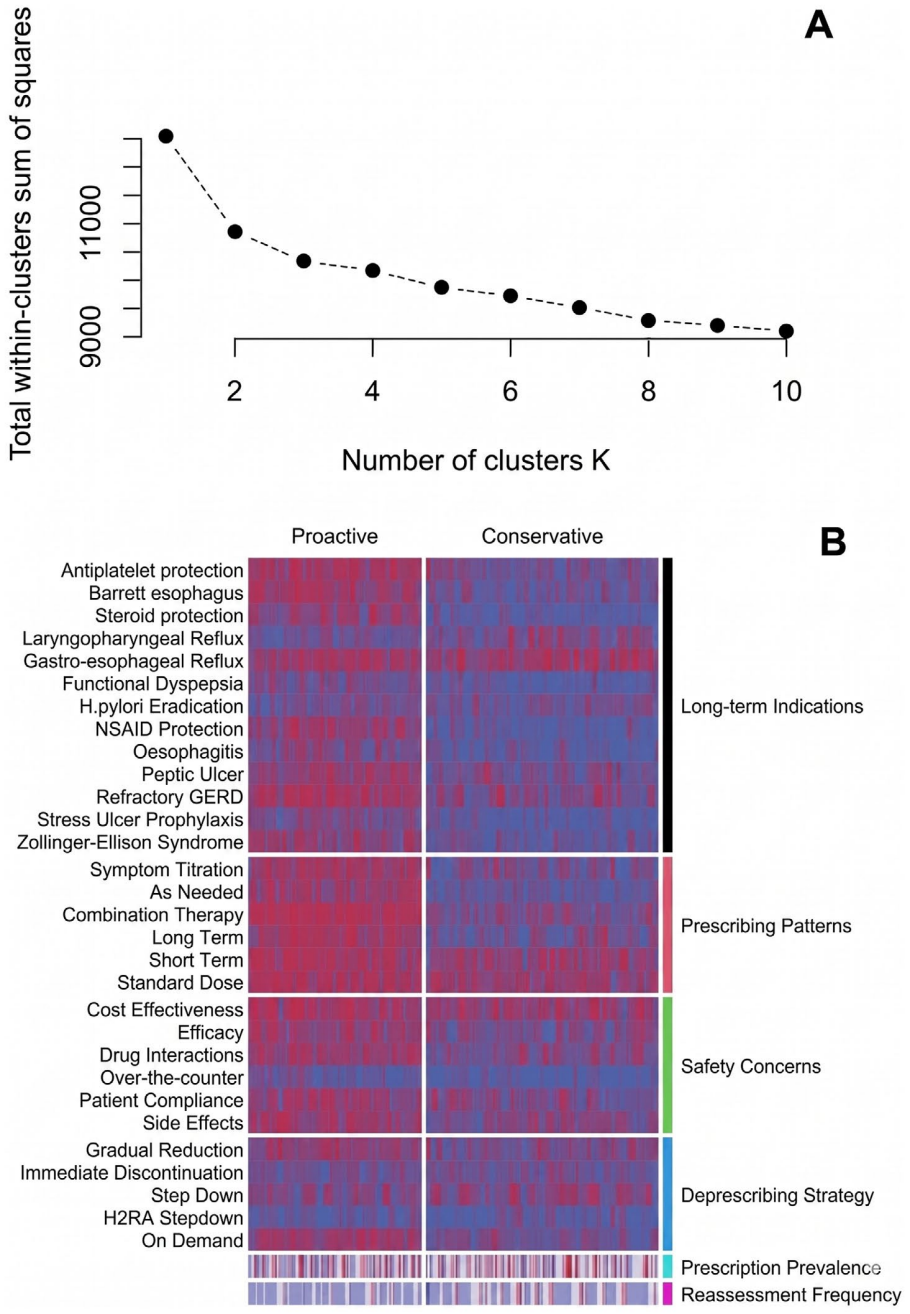
Clustering of PPI prescribing patterns. (A) The elbow plot indicates that the optimal number of clusters was 2 on the basis of within‐cluster dissimilarity via the K‐modes algorithm. (B) Heatmap visualization reveals two distinct prescribing patterns: a “Proactive” cluster characterized by frequent indication‐based prescribing, dose adjustment strategies, and diverse rationalization methods and a “Conservative” cluster characterized by limited intervention and a narrower range of prescribing behaviors. Each column represents an individual respondent; rows reflect survey items on indications, adjustment, concerns, and deprescribing strategies. Long‐term Indication: Antiplatelet protection—antiplatelet therapy‐induced gastric protection; Barrett's esophagus—Barrett's esophagus; steroid protection—chronic steroid therapy‐induced gastric protection; laryngopharyngeal reflux—extraesophageal reflux/laryngopharyngeal reflux; gastroesophageal reflux—gastroesophageal reflux disease; functional dyspepsia—functional dyspepsia; *H. Pylori* eradication— *Helicobacter pylori* eradication therapy; NSAID protection—NSAID‐induced gastric protection; oesophagitis—oesophagitis; peptic ulcer—peptic ulcer disease; refractory GERD—refractory gastroesophageal reflux disease; stress ulcer prophylaxis—stress ulcer prophylaxis; Zollinger–Ellison syndrome—Zollinger–Ellison syndrome; deprescribing strategy; symptom titration—symptom‐based titration; as needed—as‐needed basis; combination therapy—combination with other medications; long term—use beyond 8 weeks; short term—short‐term use (< 8 weeks); standard dose—guideline‐recommended dose; long‐term prescribing concerns; cost effectiveness—cost‐effectiveness; efficacy—efficacy.

The optimal number of clusters was determined to be two based on the elbow plot (Figure 2A). Heatmap visualization revealed consistent and statistically significant differences in prescribing behaviors between the two clusters (Figure 2B). The two identified clusters exhibited significant differences in guideline adherence, dosing adjustment strategies, and rationalization practices. Accordingly, they were categorized as “proactive prescribers” and “conservative prescribers” (Table S2B).

Proactive prescribers (*n* = 326, 42.7%) were characterized by a broader application of PPIs in alignment with clinical guidelines, encompassing indications such as Barrett's esophagus, NSAID‐induced gastric protection, peptic ulcer disease, and refractory GERD. They tend to employ flexible, individualized dosing approaches (e.g., symptom‐based titration, combination therapy) and routinely reassess the need for ongoing therapy at each follow‐up. Additionally, this group demonstrates greater familiarity with evidence‐based recommendations, more frequent application of rationalization strategies (e.g., gradual tapering, on‐demand use), and increased use of adjunctive therapies such as alginates tailored to the clinical context and patient preference.

In contrast, conservative prescribers (*n* = 437, 57.3%) used PPIs more restrictively, with fewer recognized indications and lower adherence to individualized or long‐term prescribing approaches. They were less likely to reassess therapy regularly and reported limited guideline‐based rationalization strategies. Familiarity with adjunctive options—such as alginate therapy for GERD management—was also notably reduced in this group. Multivariate analysis revealed several independent predictors of proactive prescribing, including medical specialty, years of clinical experience, work setting, and country (Table 6).

**TABLE 6:** Demographic and professional factors associated with proactive PPI prescription: Univariate and multivariate logistic regression analyses.

Factor	Univariate analysis			Multivariate analysis		
	OR	95% CI	*p*	OR	95% CI	*p*
Gastroenterologist	2.63	1.96, 3.53	< 0.001	2.89	2.04, 4.14	< 0.001
≥ 10 years of experience (high experience)	1.62	1.21, 2.16	0.001	1.49	1.07, 2.08	0.019
Academic/research	3.30	2.09, 5.34	< 0.001	2.42	1.44, 4.16	0.001
Hospital‐based (government)	1.09	0.82, 1.45	0.554	1.71	1.12, 2.64	0.015
Hospital‐based (private)	1.05	0.78, 1.40	0.751	1.76	1.12, 2.78	0.015
Private practice	1.55	1.09, 2.20	0.015	1.91	1.19, 3.11	0.008
Country of practice						
Vietnam	1	—	Ref	1	—	Ref
Philippines	1.70	1.13, 2.55	0.010	1.87	1.15, 3.04	0.012
Indonesia	3.05	1.74, 5.38	< 0.001	2.17	1.16, 4.07	0.015
Thailand	3.90	2.52, 6.09	< 0.001	3.74	2.30, 6.14	< 0.001
Malaysia	2.86	1.80, 4.57	< 0.001	4.84	2.81, 8.45	< 0.001
Singapore	5.94	2.43, 16.0	< 0.001	5.19	1.99, 14.8	0.001

## 4 Discussion

This study provides important insights into PPI prescribing and rationalization behaviors across the SEA region and across diverse groups of healthcare professionals including GIs, geriatric physicians and pharmacists. Using a newly developed online survey with good content validity and internal consistency, our findings revealed that while a substantial proportion of healthcare professionals adopt guideline‐concordant strategies, notable heterogeneity persists in terms of reassessment practices and rationalization approaches. In addition, using K‐mode clustering, the study identified the presence of two distinct prescriber profiles among the participants, that is, the proactive versus conservative prescribers.

Regarding typical prescription of PPIs in the SEA, it is encouraging to note that the majority of participants reported using standard, guideline‐recommended dosages and limiting therapy to short‐term duration based on the clinical indications (70.1% and 68.9%, respectively). However, there is a notable difference in the use of long‐term PPIs depending on the specialty of the survey participants: GIs are more likely than non‐GIs to prescribe long‐term PPIs for conditions supported by guideline‐based indications, such as Barrett's esophagus and antiplatelet‐induced and NSAID‐induced gastric protection. They were also significantly more likely than non‐GIs to apply symptom‐based titration and on‐demand use. This nuanced approach may reflect the GIs' greater familiarity with PPI pharmacodynamics and more comfort in tailoring therapy based on individual response, as supported by previous studies [14, 15, 16]. However, regarding long‐term use of PPIs, our study showed that there were potentially inappropriate indications, especially for managing GERD (67.8%), peptic ulcer disease (43.8%) and preventing steroid‐induced gastrointestinal injury (30.7%). GERD is typically mild and rarely presents with complications in Asia [8, 17]. Therefore, the latest SEA guideline recommends on‐demand PPI therapy for nonerosive or mild erosive reflux disease, as it is as effective as continuous therapy [15, 17, 18]. Similarly, peptic ulcer healing generally requires only 4–8 weeks of PPI treatment unless *H. pylori* eradication is unsuccessful or when aspirin/NSAID use must be continued [9, 15, 19, 20]. Although chronic steroid use has historically prompted gastroprotection, current guidelines worldwide suggest that PPI prophylaxis should be reserved for patients with increased gastrointestinal risk who are on antiplatelet or NSAID therapy, and not steroids alone [9, 15, 16]. Interestingly, this practice of PPI prophylaxis was more commonly reported among HE prescribers which may reflect delayed adoption of evolving recommendations among HE prescribers, highlighting the importance of targeted continuing medical education.

While concerns regarding PPI overuse are well recognized, our findings also suggest that underutilisation for certain evidence‐based indications may represent an additional and important dimension of inappropriate prescribing. Notably, the relatively low proportions of respondents reporting PPI use for NSAID‐related gastroprotection (33.7%) and Barrett's esophagus (43.9%) may indicate potential gaps in adherence to guideline‐recommended practices.

Previous real‐world studies have similarly reported suboptimal use of gastroprotective strategies among patients at increased risk of NSAID‐related gastrointestinal complications [21]. However, these findings should be interpreted with caution. Current and previous clinical guidance recommend that PPI prophylaxis for NSAID users should be reserved for patients with established gastrointestinal risk factors rather than routinely prescribed to all NSAID users, as outlined in earlier guidelines and reinforced by more recent literature on appropriate gastroprotection strategies [22, 23]. In addition, our survey did not capture detailed patient‐level risk stratification; therefore, lower reported rates of PPI use may, in part, reflect appropriate clinical practice. Likewise, although PPI therapy is frequently used in patients with Barrett's esophagus, its role in preventing progression to dysplasia or esophageal adenocarcinoma remains supported by limited or conditional evidence in contemporary guidelines [24, 25].

Taken together, these findings highlight the complexity of PPI prescribing in clinical practice, where both overuse and potential underuse may coexist, and should be addressed through nuanced stewardship strategies rather than a singular focus on deprescribing.

Reassessment of PPI therapy is an essential practice to ensure continued clinical justification and to prevent inappropriate long‐term use. In our study, most healthcare professionals (76%) reported reassessing PPI indications either at every follow‐up visit or at regular intervals of 1–3 months. This frequency is consistent with international guidelines, particularly when PPI use extends beyond the initial treatment phase [9, 14, 15]. Our findings also revealed a wide range of approaches to PPI deprescribing, with most clinicians preferring structured, stepwise strategies over abrupt discontinuation. The latest AGA guidelines suggest that either dose tapering or abrupt discontinuation of PPIs be considered when deprescribing PPIs, given that patients should be advised to be mindful of developing recurrent upper gastrointestinal symptoms as a consequence of rebound acid hypersecretion [14]. In this context, alginate therapy may serve as an effective rescue option to manage breakthrough symptoms. Mechanistically, alginates form a viscous “raft” that floats on top of the gastric contents, acting as a physical barrier to prevent gastroesophageal reflux without inhibiting acid secretion—making them particularly suitable during PPI tapering [26, 27]. Our findings reflect a generally high level of awareness of PPI stewardship across clinical domains. Nonetheless, the remaining surveyed healthcare professionals admitted to less frequent reassessment, raising concerns about missed opportunities for deprescribing.

From the K‐mode cluster analysis, more than half of the survey participants classified as conservative PPI prescribers underscored the importance and urgency of developing a regional practice guideline tailored to the specific needs and epidemiological context of the SEA region. Specifically, there were statistically significant differences in the proportion of conservative prescribers across countries. With respect to medical specialties, GIs were more likely to adopt proactive approaches than the non‐GIs, and HE providers were more frequently proactive than their LE counterparts. A recent study conducted in Malaysia investigating factors influencing healthcare providers' behaviors in deprescribing revealed that their knowledge and clinical experience played pivotal roles in shaping decisions to reduce or discontinue medications [28]. However, this study addressed general attitudes toward deprescribing without focusing specifically on PPIs. Our findings corroborate these results within the specific context of PPI use. A recent systematic review highlighted various strategies can effectively support PPI deprescription, including interprofessional collaboration, the application of clinical decision‐making algorithms, and active patient involvement [29]; hence, our findings contribute to identifying target subgroups of healthcare providers that should be prioritized to improve the appropriate prescribing and deprescribing of PPIs.

An important strength of our study lies in its focus on healthcare professionals' perspectives regarding PPI use and rationalization, which is based on a relatively large and diverse sample. This approach provides a broader understanding than prior studies in the region, which have primarily relied on prescription audits. Additionally, the validated survey and clustering analysis yield robust insights into prescriber behavior, offering context‐specific guidance for policy and clinical practice. However, this study has several limitations. First, its generalizability is constrained by the fact that participants were drawn from only six of the 10 countries in the SEA region. Second, the unequal distribution of respondents across countries may introduce bias and limit the representativeness of PPI prescribing and rationalization practices across the region. These limitations should be considered when interpreting and applying the findings to regional policy development. Third, this study relied on self‐reported survey responses, which may be subjected to recall or social‐desirability bias. To address this, future studies could incorporate objective data, such as prescription records, to validate findings. Forth, the pilot sample was selected using a convenience approach and was not intended to be representative of the target population; however, its role was limited to assessing questionnaire clarity and usability. Finally, the distribution of respondents across countries was uneven, with particularly high participation from Vietnam and low participation from Singapore. This likely reflects differences in survey uptake rather than true differences in prescribing practices, as participation did not consistently align with country population size. As a result, the findings should not be considered representative at the country level, and comparisons between countries should be interpreted with caution. Furthermore, variations in healthcare system characteristics across the region may influence prescribing behaviors; however, these factors were not captured in this survey. Therefore, the findings should be interpreted primarily as a regional overview rather than country‐specific estimates.

In conclusion, this study revealed marked variability in PPI prescribing and rationalization practices across the SEA region. It also identified several indications where PPI use is likely inappropriate, both in terms of clinical justification and prolonged duration of therapy. Most importantly, the study characterized a distinct group of conservative prescribers, who accounted for more than half of the survey respondents. These findings underscore the urgent need to establish a regional consensus to promote evidence‐based clinical practice. The study may also provide an important foundation for designing targeted, context‐specific interventions aimed at minimizing the inappropriate and unnecessarily prolonged use of PPIs.

## Author Contributions

**Wee Kooi Cheah:** investigation, writing – review and editing. **Rahela Ambaras Khan:** investigation, writing – review and editing. **Sakkarin Chirapongsathorn:** investigation, writing – review and editing. **Kalwinder Singh Khaira:** investigation, writing – review and editing. **Augusto Jose G. Galang:** investigation, writing – review and editing. **Andrew Ming‐Liang Ong:** investigation, writing – review and editing. **Phuripong Kijdamrongthum:** investigation, writing – review and editing. **Tju Siang Chua:** investigation, writing – review and editing. **Tien Manh Huynh:** methodology, validation, data curation, formal analysis, software, writing – review and editing. **Hang Viet Dao:** investigation, writing – review and editing. **Yeong Yeh Lee:** project administration, supervision, investigation, resources, funding acquisition, writing – review and editing. **Duc Trong Quach:** conceptualization, methodology, validation, data curation, formal analysis, investigation, writing – original draft, writing – review and editing. **Jose Sollano:** investigation, writing – review and editing. **So Fie Tan:** investigation, writing – review and editing. **Somchai Leelakusolvong:** project administration, supervision, investigation, resources, funding acquisition, writing – review and editing. **Ari Fahrial Syam:** investigation, writing – review and editing.

## Funding

This research was financially supported by Reckitt Benckiser (Singapore) Pte Ltd., which provided funding exclusively for the article processing charge. The sponsor had no involvement in the conception or design of the study, the acquisition, analysis, or interpretation of data, the drafting of the manuscript, or the decision to submit it for publication.

## Ethics Statement

Informed consent to participate was implied by the completion and submission of the anonymized online survey by the healthcare professionals, as approved by the relevant Ethics Committee/Institutional Review Board.

## Consent

The authors have nothing to report.

## Conflicts of Interest

The authors declare no conflicts of interest.

## Supporting information


**Data S1:** Survey contents.

**Table S1:** Frequency of reassessing the indication for PPI use.

**Table S2:** (A) Comparative characteristics of proactive and conservative PPI prescribing patterns. (B) Distribution of prescribing patterns by demographic and professional characteristics.

## Data Availability

De‐identified individual participant data and the study protocol will be available upon reasonable request to the corresponding author. Data access will be granted solely for research purposes and in accordance with ethical approvals and applicable data protection regulations.
