# Supplementary material for: Proton Pump Inhibitors Prescribing Behaviors and Rationalization Strategies Among Healthcare Providers in Southeast Asia
Source: Pharmacol Res Perspect. 2026 Jun 2;14(3):e70274. doi: 10.1002/prp2.70274 (PMC13239532; doi:10.1002/prp2.70274)
Supplement: Supplementary file 1 — Data S1: Survey contents. [file PRP2-14-e70274-s002.docx]

**Supplement 1.** Survey contents

## **Section 1: General Information**

1. **Specialty:**
   - Gastroenterology and hepatology
   - General practitioner/family physician
   - Geriatric medicine
   - Internal medicine
   - Otolaryngology
   - Pharmacist
   - Other (please specify): _________
2. **Years in practice:**
   - < 5 years
   - 5–10 years
   - 11–20 years
   - > 20 years
3. **Practice setting: (please select all that apply)**
   - Private practice
   - Hospital-based (private)
   - Hospital-based (government)
   - Academic/research
   - Other (please specify): _________
4. **Country of practice:**
   - Indonesia
   - Malaysia
   - Philippines
   - Singapore
   - Thailand
   - Vietnam
   - Other (please specify): ________

## **Section 2: PPIs Prescribing Habits**

1. **What are your common indications for prescribing or recommending PPIs long-term?**
   **(Please select all that apply)**

□ Antiplatelet therapy-induced gastric protection

□ Barrett’s oesophagus

□ Chronic steroid therapy-induced gastric protection

□ Extraesophageal reflux/laryngopharyngeal reflux (LPR)

□ Gastroesophageal reflux disease (GERD)

□ Functional dyspepsia

□ *Helicobacter pylori* eradication therapy

□ Nonsteroidal anti-inflammatory drug (NSAID)-induced gastric protection

□ Oesophagitis

□ Peptic ulcer disease

□ Refractory gastroesophageal reflux disease (GERD)

□ Stress ulcer prophylaxis in critically ill patients

□ Zollinger–Ellison syndrome

□ Other (please specify): _________

1. **How do you typically prescribe or recommend PPIs for your patients?** **(Please select all that apply)**

□ Adjustable dosage titrated on the basis of symptom severity or response

□ As-needed basis when symptoms arise

□ Combination therapy with other medications (e.g., *H. pylori* eradication)

□ Long-term use (beyond 8 weeks) for chronic conditions or maintenance therapy

□ Short-term use (< 8 weeks) for a defined duration on the basis of indication

□ Standard, guideline-recommended dosage for a specific PPI

□ Other (please specify): _________

1. **What are your concerns when prescribing or recommending PPIs for your patients long-term? (Please select all that apply)**

□ Cost-effectiveness

□ Drug interactions

□ Efficacy

□ Over-the-counter indications

□ Patient compliance

□ Side effects

□ Other (please specify): _________

1. **What percentage of your prescriptions in the past month included PPIs?**

- ≤ 10%
- 11–20%
- 21–30%
- 31–40%
- ≥ 50%

## **Section 3: PPI rationalisation strategies**

1. **How frequently do you reassess the indication for PPI use in patients currently taking the medication?**
   - Annually
   - At every follow-up visit
   - Every 1–3 months
   - Every 3–6 months
   - Only when new symptoms or concerns arise
   - Rarely or never
   - Other (please specify): _________
2. **What is your typical strategy for rationalising PPIs when they are no longer needed or indicated? (Please select all that apply)**

- Gradual dose reduction over time
- Immediate discontinuation of PPIs
- Step down to antacids or alginates
- Step-down to H2 receptor antagonists (H2RAs)
- Switching to on-demand PPI use
- Other (please specify): _________

1. **How often do you consider using alginate as part of your PPI rationalisation strategy for patients with GERD in your real-life practice?**

- Always
- Often
- Occasionally
- Rarely
- Never

1. **What are the key factors to consider when deciding to use alginate as part of a PPI rationalisation strategy in GERD management? (Please select all that apply)**

□ Availability and cost of alginate therapy

□ Evidence supporting its efficacy in mild-to-moderate GERD

□ Patient preference for nonsystemic/nonpharmaceutical drugs

□ PPI tolerance or contraindications

□ Recommendations from clinical guidelines

□ Risk of rebound acid hypersecretion during PPI tapering

□ Other (please specify): _______________

1. **What are the reasons for not using alginate as part of a PPI rationalisation strategy in GERD management? (Please select all that apply)**

□ Accessibility issues

□ Concerns about patient adherence to alginate therapy

□ Cost

□ Lack of evidence supporting its efficacy

□ Limited familiarity with alginate as a treatment option

□ Perceived ineffectiveness compared with other therapies

□ Preference for other treatment options (e.g., H2RA)

□ None of the above difficulties encountered

□ Other (please specify): _________
