# Supplementary material for: Proton Pump Inhibitors Prescribing Behaviors and Rationalization Strategies Among Healthcare Providers in Southeast Asia
Source: Pharmacol Res Perspect. 2026 Jun 2;14(3):e70274. doi: 10.1002/prp2.70274 (PMC13239532; doi:10.1002/prp2.70274)
Supplement: Supplementary file 2 — Table S1: Frequency of reassessing the indication for PPI use. [file PRP2-14-e70274-s003.docx]

**Supplementary Table 1.** Frequency of reassessing the indication for PPI use

| **Frequency** | **All**  **n=763**  **(%)** | **Speciality** | | **p*** | **Experience** | | **p*** |
| --- | --- | --- | --- | --- | --- | --- | --- |
|  |  | **Non-GI**  **n=386**  **(%)** | **GI**  **n=377**  **(%)** |  | **LE**  **n=394**  **(%)** | **HE**  **n=369**  (%) |  |
| At every follow-up visit | 452 (59.2) | 232  (60.1) | 220  (58.4) | 0.585 | 245  (62.2) | 207  (56.1) | 0.162 |
| Every 1-3 months | 128 (16.8) | 63  (16.3) | 65  (17.2) |  | 61  (15.5) | 67  (18.2) |  |
| Every 3-6 months | 70  (9.2) | 31  (8.0) | 39  (10.3) |  | 37  (9.4) | 33  (8.9) |  |
| Annually | 20  (2.6) | 10  (2.6) | 10  (2.7) |  | 13  (3.3) | 7  (1.9) |  |
| Only when new symptoms or concerns arise | 82 (10.7) | 42  (10.9) | 40  (10.6) |  | 34  (8.6) | 48  (13.0) |  |
| Rarely or never | 11  (1.4) | 8  (2.1) | 3  (0.8) |  | 4  (1.0) | 7  (1.9) |  |

GI – Gastroenterologist; Non-GI – Non-Gastroenterologist; HE – High Experience; LE – Low Experience. (*) Pearson’s chi-square test was used to compare proportions (Fisher’s exact test was applied where appropriate).
